# Overexpressing Kallistatin Aggravates Experimental Autoimmune Uveitis Through Promoting Th17 Differentiation

**DOI:** 10.3389/fimmu.2021.756423

**Published:** 2021-10-18

**Authors:** Nu Chen, Shuang Chen, Zhihui Zhang, Xuexue Cui, Lingzi Wu, Kailei Guo, Hui Shao, Jian-Xing Ma, Xiaomin Zhang

**Affiliations:** ^1^ Tianjin Key Laboratory of Retinal Functions and Diseases, Tianjin Branch of National Clinical Research Center for Ocular Disease, Eye Institute and School of Optometry, Tianjin Medical University Eye Hospital, Tianjin, China; ^2^ Department of Ophthalmology and Visual Sciences, Kentucky Lions Eye Center, University of Louisville, School of Medicine, Louisville, KY, United States; ^3^ Department of Physiology, University of Oklahoma Health Sciences Center, Oklahoma City, OK, United States

**Keywords:** kallistatin, uveitis, experimental autoimmune uveitis, autoimmune disease, immunology, interphotoreceptor retinoid-binding protein, Th17

## Abstract

Kallistatin or kallikrein-binding protein (KBP) has been reported to regulate angiogenesis, inflammation and tumor progression. Autoimmune uveitis is a common, sight-threatening inflammatory intraocular disease. However, the roles of kallistatin in autoimmunity and autoreactive T cells are poorly investigated. Compared to non-uveitis controls, we found that plasma levels of kallistatin were significantly upregulated in patients with Vogt-Koyanagi-Harada (VKH) disease, one of the non-infectious uveitis. Using an experimental autoimmune uveitis (EAU) model induced by human interphotoreceptor retinoid-binding protein peptide 651-670 (hIRBP_651-670_), we examined the effects of kallistatin on the pathogenesis of autoimmune diseases. Compared to wild type (WT) mice, kallistatin transgenic (KS) mice developed severe uveitis with dominant Th17 infiltrates in the eye. In addition, the proliferative antigen-specific T cells isolated from KS EAU mice produced increased levels of IL-17A, but not IFN-γ or IL-10 cytokines. Moreover, splenic CD4^+^ T cells from naïve KS mice expressed higher levels of Il17a mRNA compared to WT naïve mice. Under Th17 polarization conditions, KS mice exhibited enhanced differentiation of naïve CD4^+^ T cells into Th17 cells compared to WT controls. Together, our results indicate that kallistatin promotes Th17 differentiation and is a key regulator of aggravating autoinflammation in EAU. Targeting kallistatin might be a potential to treat autoimmune disease.

## Introduction

Autoimmune uveitis or non-infectious uveitis (NIU) is a group of intraocular inflammatory disease, which can affect only the eyes, or it can be part of a systemic disease, and Vogt-Koyanagi-Harada (VKH) is one of NIU (1). Uveitis, if untreated, can cause a significant visual deficit and even blindness, which accounts for 10-15% of cases of severe visual impairment in the developed countries (2, 3). Due to easy recurrence, more frequent occurrence in younger adults (4), autoimmune uveitis can cause heavy social and economic burdens. However, the pathogenesis of autoimmune uveitis remains unknown. Traditional therapeutic drugs, such as corticosteroids and immunosuppressants, when used for long periods of time, can yield a spectrum of local or systemic side effects. Although the new biological agents are more targeted, there are still systemic side effects that are insurmountable (5, 6). Thus, there is an urgent need to study pathological mechanisms of the autoimmune uveitis and explore effective drugs with strong pertinence and safety to treat autoimmune uveitis.

Studies in experimental autoimmune uveitis (EAU), animal models for autoimmune panuveitis or posterior uveitis in the human have learned that autoreactive CD4^+^ T cells play an important role in ocular inflammation (7–9). Antigen-responsive CD4^+^ T cells from EAU mice transferred into naïve mice initiate autoimmune uveitis, and different effector T cells can induce experimental models with different pathological phenotypes (10–12). Interleukin 17 (IL-17) producing Th17 cells and IFN-γ-producing Th1 cells, have been thought to be important in the immunopathogenesis of uveitis and EAU (8, 13). The IFN-γ^+^ IL-17A^+^ phenotype, named Th1/Th17 subset, is associated with Th17-mediated diseases in human and animal models (14). And it has been recently shown that CD4^+^ T cells or Th17 cells have substantial plasticity and display important functions in the tissue microenvironment during chronic inflammation (14–17). Nevertheless, the mechanisms by which these autoreactive T cells regulate the autoimmune response during EAU are not fully elucidated.

Kallistatin, also named SERPINA4, was originally identified as a tissue kallikrein-binding protein (KBP), a member of serine protease inhibitor (serpin) family (18–20). It has been previously reported that kallistatin exhibits various functions and protective effects in disease models of superficial angiogenesis or acute inflammation, such as arthritis, septic shock, renal injury, myocardial ischemia, hypertension and diabetic retinopathy (21–26). Increased circulating kallistatin levels have been observed in diabetic patients with microvascular complications (27, 28). And elevated kallistatin in diabetes has been suggested to promote the recruitment of macrophages and M1 polarization, aggravating the inflammatory responses and impairing the healing of diabetic wound, a chronic inflammation (29). All these findings suggest a potential role of kallistatin in modulating inflammation and immune system. However, how kallistatin affects autoimmune mediated inflammation remains poorly understood, and the roles of kallistatin in adaptive immune cells such as Th1 and Th17 cells have not yet been studied.

The primary aim of this study was to elucidate the roles of kallistatin in the pathogenesis of autoimmunity. We first detected the expression levels of kallistatin in the plasma of patients with VKH and controls. We additionally used kallistatin-transgenic (KS) mice and wild-type (WT) mice to induce EAU with human interphotoreceptor retinoid-binding protein (IRBP) peptide 651-670 (hIRBP_651-670_), and compared the severity of EAU by various ocular imaging techniques and pathological verification. We also determined whether and how kallistatin overexpression regulates T-cell mediated autoimmune responses. Our results suggest that kallistatin overexpression enhances Th17 differentiation and exacerbates intra-ocular inflammation during EAU. Consequently, it provides interesting ways to think about therapeutic avenues for autoimmune diseases, such as uveitis.

## Materials and Methods

### Human Subjects and Sample Collection

The studies involving human participants were reviewed and approved by the Ethics Committee of Tianjin Medical University Eye Hospital, Tianjin, China (No. 2016KY-14). All participants provided their written informed consent in this study. All VKH patients with initial onset, significant ocular inflammation and no treatments of systemic corticosteroids were diagnosed and recruited by ophthalmologist.

### Animals

C57BL/6J (B6) female wild-type (WT) mice were purchased from Vital River Laboratory Animal Technology (Beijing, China). The kallistatin-transgenic mice with same background of B6 were provided as a gift from Dr. Jianxing Ma (University of Oklahoma Health Sciences Center), and the transgene expression was confirmed by real-time PCR (RT-PCR) of spleen and draining lymph nodes (dLNs) RNA using the genotyping primers. Mice were bred and maintained under specific-pathogen-free conditions. Animal care and involving experiments were performed according to the guidelines approved by the Animal Care and Use Committee of the Tianjin Medical University, and mice were used at 6 to 10 weeks of age.

### LC-MS/MS Analysis for Proteomics

Samples were collected and prepared for LC-MS/MS analysis. Briefly, the plasma samples were lysed by 8M urea lysate. After reduction and alkylation, samples were trypsinized with sequencing grade modified trypsin (Promega, USA) for 16 h at 37°C to get the peptide segments. Plasma-derived tryptic peptide fragments were eluted with 0.1% FA and 40% CAN buffer and dried *via* Integrated SpeedVac (Thermo Fisher, USA). Peptide mixtures were loaded onto a self-packed trap column (100μm×2cm, Durashell C18 3 μm, 120A˚), separated on a homemade analytical C18 column (150μm×15cm, 1.9 μm), and monitored on an electrospray-ionization Triple TOF 6600 mass spectrometers (AB SCIEX, USA). The elution was performed over a gradient 120 min with buffer B ranging from 5% to 80%.

### EAU Induction

Mice were injected subcutaneously with 250 μg of human interphotoreceptor retinoid binding protein peptide [LAQGAYRTAVDLESLASQLT (hIRBP_651-670_), Shanghai Hanhong Chemical Co., Ltd., Shanghai, China] emulsified 1:1 v/v in complete Freund’s adjuvant (CFA, Sigma Aldrich) containing 3.5 mg/mL mycobacterium tuberculosis H37RA (BD Biosciences). A total of 200 μL emulsion was distributed along the base of the tail (100 μL) and both thighs (50 μL each). In all immunizations, 500 ng pertussis toxin (List Biological Laboratories Inc.), per mouse was injected intraperitoneally (i.p.) immediately on the day of immunization and the following day.

### Clinical and Histopathological Scoring

Clinical disease was scored at regular intervals (3 times a week) by indirect fundoscopy (Hein, Germany). The clinical signs of EAU were assessed with a 0–4 scoring system, according to the criteria previously published by Caspi’s group (30). In brief, the clinical scale is as follows: 0 = No inflammation; 0.5 = trace disease, 1 to 2 very small, peripheral focal lesions; 1 = minimally active, localized, <5 focal lesions; ≤1 linear lesion; 2 = moderately active, multiple (>5) lesions; severe vasculitis; <5 linear lesions; 3 = Active, multiple diffuse lesions; 4 = very active, large retinal detachment or hemorrhage. For histology, animals were sacrificed 21 days after immunization, eyes were surgically resected, fixed in formalin for 24 h, and embedded in paraffin. Then 4-μm-thick sections of the pupil and optic nerve axis were made and stained with hematoxylin-eosin (H&E) staining. The retinal histopathological changes were also graded on a scale of 0–4, according to previously published criteria (30).

### Retinal Imaging Evaluations

Retinal imaging evaluations were conducted with spectral-domain optical coherence tomography (SD-OCT, Heidelberg Engineering, Heidelberg, Germany) and retinal imaging microscope (Micron IV; Phoenix Research Labs, Pleasanton, CA, USA). ImageJ software (National Institutes of Health, USA) was used to quantify the numbers of hyperreflective foci in the vitreous cavity of the SD-OCT image. The fundus images under visible light were collected by retinal imaging microscope.

### Isolation of Infiltrating Cells From Eyes

The eyes of mice were collected on the indicated days after immunization. After removal of the cornea, lens, optic nerve and excess connective tissue, the rest of the eyes was ground up on the steel screen, followed by digestion with RPMI1640 containing 1 mg/ml collagenase D (Roche) for 1 h at 37°C, a shaking speed of 220 rpm. After passing through the 70μm strainer, the single cell suspension from inflamed eyes was collected.

### Flow Cytometry Analysis

Aliquots of single cells (1 × 10^6^) were stained with APC-labeled anti-mouse CD4 (BioLegend, Cat# 100412) antibodies or CD25 (BioLegend, Cat# 101908), CD62L (BioLegend, Cat# 104407), CD44 (BioLegend, Cat# 103035) or other appropriately labeled antibodies. Samples were then washed and resuspended with staining buffer [PBS containing 5% fetal bovine serum (FBS)], followed by immediately flow analysis. For intracellular staining, cells were further fixed and permeabilized with fixation/permeabilization buffer (BioLegend). Intracellular staining was performed with FITC-conjugated anti-mouse IFN-γ (BioLegend, Cat# 505806) antibodies or PE-labeled anti-mouse IL-17A antibodies (BioLegend, Cat# 506908) in permeabilization wash buffer. Flow cytometric analysis was performed on flow cytometry (FACS_calibur_, BD Biosciences).

### Assays for *Ex Vivo* T Cell Recall and IRBP-Specific T Cell Proliferation

T cells from spleens of WT or KS mice were isolated on day 14-16 after immunization using nylon wool column and stimulated at 4 × 10^5^ cells/well with different concentrations of IRBP_651-670_ in the presence of 1 × 10^5^ irradiated syngeneic APCs in 96-well plates. The cells were cultured in the incubator for 60 hours, and BrdU was incorporated into the culture medium at the last 16 hours to evaluate cell proliferation, following the manufacturer’s protocol of BrdU assay kit (Millipore, 11647229001). Secreted IL-17A, IFN-γ and IL-10 cytokines in the supernatants were determined using ELISA kits according to the manufacturer’s instructions (R&D Systems).

### RNA Isolation, Reverse Transcription of cDNA, and Real-Time Quantitative PCR

Cells or tissue samples from the spleen, dLNs, and eye were homogenized and lysed with 1ml TRIzol (Ambion, 15596018). Total RNA was reverse transcribed to cDNA using the superscript First-Strand Synthesis System and random hexamer primers (Thermo Fisher Scientific, K1622), according to the manufacturer’s instructions. The cDNA was then used as a template for real-time PCR using SYBER Green Master Mix (Roche, 41472600) and gene specific primers. For each sample, the transcript copy numbers were normalized to housekeeping gene glyceraldehyde phosphate dehydrogenase (*Gapdh*), and the fold induction compared to the control was calculated. The following gene-specific primers were used for analysis:

*SERPINA4*: F-AGGGAAGATTGTGGATTTGG, R-ATGAAGATACCAGTGATGCTC;
*Gapdh*: F-CCTGTTGCTGTAGCCGTATTCA, R-CCAGGTTGTCTCCTGCGACTT;
*Il17a*: F-CTGGAGGATAACACTGTGAGAGT, R-TGCTGAATGGCGACGGAGTTC;
*Il17f*: F-GAGGATAACACTGTGAGAGTTGAC, R-GAGTTCATGGTGCTGTCTTCC;
*Il21*: F-TCATCATTGACCTCGTGGCCC, R-ATCGTACTTCTCCACTTGCAATCCC;
*Il22*: F-CATGCAGGAGGTGGTACCTT, R-CAGACGCAAGCATTTCTC AG;
*Il23*: F-AATAATGTGCCCCGTATCCA, R-AGGCTCCCCTTTGAAGATGT;
*Ifng*: F-GATGCATTCATGAGTATTGCCAAGT, R-GTGGACCACTCGGATGAGCTC;
*Tnfα*: F-ATCATCTTCTCAAAATTCGAGTGA, R-TTGAGATCCATGCCGTTGG;
*Gmcsf*: F-TTTACTTTTCCTGGGCATTG, R-TAGCTGGCTGTCATGTTCAA;
*Foxp3*: F-GGCCCTTCTCCAGGACAGA, R-GCTGATCATGGCTGGGTTGT;

### *In Vitro* T Cell Activation

CD4^+^ T cells were isolated from single-cell suspensions of spleen and dLNs using a mouse CD4^+^ T cell isolation kit (Invitrogen, 11415D), according to the manufacturer’s instructions. 4 × 10^5^ purified T cells were seeded in 96-well plates pre-coated with 5 μg/ml of anti-CD3 mAb (Bio X Cell, BP0001-1) overnight in the presence of 1 μg/ml of anti-CD28 mAb (Bio X Cell, BP0015-1) and 1,000 U/ml of recombinant mouse IL-2 (R&D, 402-ML). T cells were collected and stained with PE-labeled CD69 (BioLegend, Cat# 104507), followed by flow cytometry analysis.

### Th17 Differentiation Assay

Naïve CD4^+^CD25^-^62L^hi^CD44^lo^ T cells were isolated from pooled single-cell suspensions of spleen and dLNs by a BD FACS Aria III flow cytometer (BD Biosciences). Purified naïve T cells were stimulated with plate-bound anti-CD3 mAb (R&D) for 5 days in the presence of polarizing cytokines and blocking antibodies, according to the manufacturer’s protocol of the mouse Th17 cell differentiation kit (R&D, Cat# CDK017). We cultured 1.25 × 10^5^ cells/well in 96-well plates with a total volume of 0.2 ml/well of culture medium. The mouse Th17 differentiation media were refreshed on day 3 by adding an equal volume of Th17 differentiation fresh media. The IL17A productions in the supernatants were measured by ELISA following the manufacturer’s instructions.

### Statistical Analysis

We performed the statistical analyses with Prism 8 (GraphPad software). The two-tailed student’s t test was applied for the statistical comparison between two groups, and one-way ANOVA was used for three or more sets of data. The clinical score and T cell proliferation was analyzed by repeated measurement ANOVA (two-way ANOVA), using mixed models. Post-ANOVA comparisons were made using the Sidak correction. The data were expressed as means ± SEM. The p value less than 0.05 was considered statistically significant.

## Results

### Elevated Expression of Kallistatin Protein in VKH Patients

**Figure 1:**
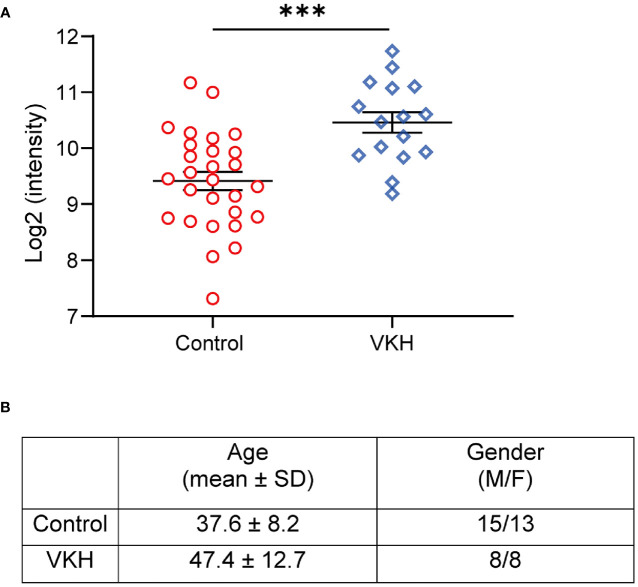
Proteomics analysis of VKH patients identifies upregulation of kallistatin protein. The 48 participants with primary diagnosis (Control: n = 28; VKH: n = 16) were enrolled in the proteomics analysis. **(A)** The plasma levels of kallistatin protein in controls and VKH patients were analyzed by mass spectrometry. **(B)** Demographics of VKH patients and control participants. The mass spectrometry data were log2 transformed and quantile normalized using the R programming language package. Differential analysis of protein expression was performed using t test. Data are shown as mean ± SEM. ***P < 0.001.

To evaluate the levels of kallistatin in uveitis patients, we collected the plasma samples from 16 VKH patients and 28 non-uveitis controls, and determined the expression of kallistatin protein by proteomics analysis. The demographics of VKH patients and control subjects were summarized, as shown in **Figure 1B**. In the proteomics data, the proteins with a difference >1.5-fold and p value < 0.05 were considered differentially expressed proteins (DEPs), and kallistatin protein was identified as one of them. Compared to the non-uveitis controls, kallistatin proteins were highly expressed with a log2 fold change 2.07 in the VKH samples (**Figure 1A**, p = 0.0002). The proteomics analysis indicated that kallistatin was overexpressed in the plasma of VKH patients.

### Kallistatin-Transgenic Mice Develop Severe EAU

**Figure 2:**
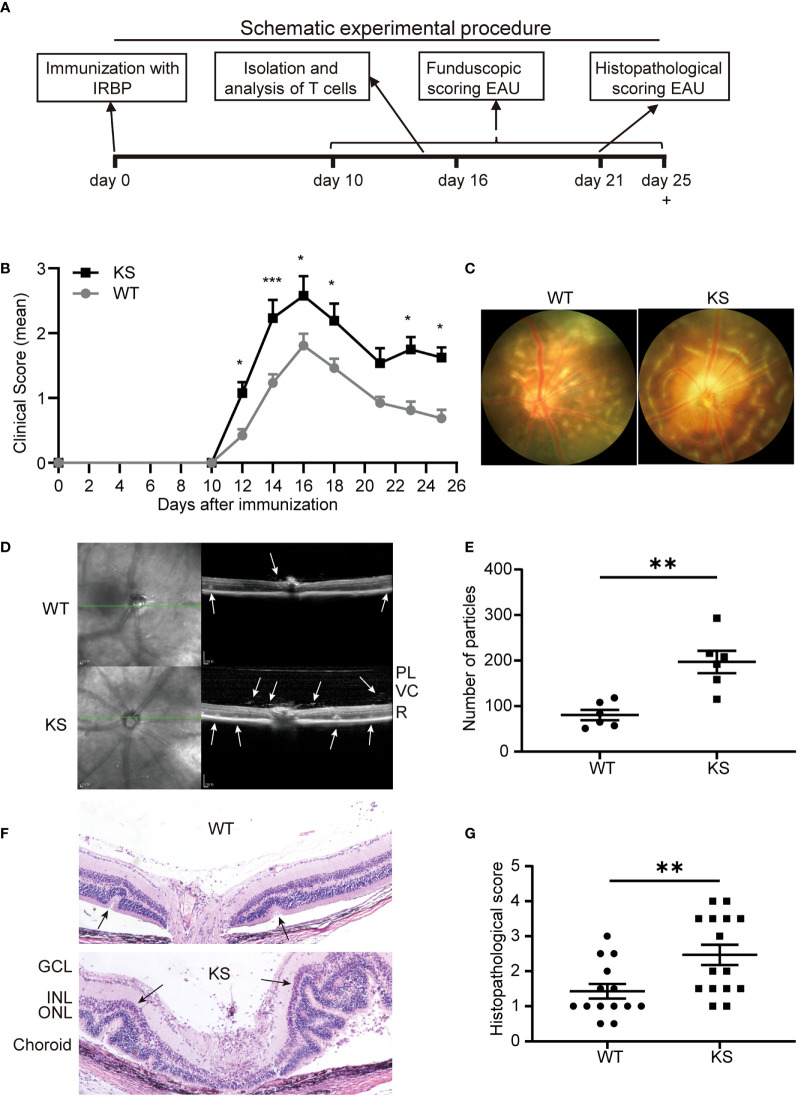
Kallistatin-transgenic (KS) mice develop exacerbated EAU. **(A)** A schematic experimental procedure of EAU induction and examination. **(B)** EAU clinical score of WT and KS mice (n = 13 in each group). Age- and sex-matched WT and KS mice were immunized with hIRBP_651-670_, and their eyes were examined by indirect ophthalmoscope and the clinical score was recorded from day 10 to day 25. Data are shown as mean ± SEM. *p < 0.05, **p < 0.01, ***P < 0.001, two-way ANOVA. **(C)** Representative images of the eyes in WT and KS mice on day 16 after EAU induction by retinal imaging microscope. **(D)** Representative images showing fundus condition by SD-OCT. Hyper-reflective foci in the vitreous cavity and retinal folds (white arrows) were observed around the optical nerve. PL, posterior lens margin; VC, vitreous cavity; R, retina. **(E)** Particles in the SD-OCT images were assessed and calculated using ImageJ. **(F)** Pathological representatives of hematoxylin and eosin (H&E) staining of the eye sections from WT and KS mice on day 21 after immunization. The retinal folds and protrusions near the optic disk were indicated by the black arrows. GCL, ganglion cell layer; INL, inner nuclear layer; ONL, outer nuclear layer. Original magnification, 20X. **(G)** Histopathological scores for EAU in WT and KS mice at day 21 post immunization. Data are presented as the mean ± SEM. N = 6 **(E)** or > 6 **(G)**; **p < 0.01, Mann-Whitney test.

To investigate whether overexpressing kallistatin affects autoimmunity such as uveitis, we induced EAU with hIRBP_651-670_ in WT and kallistatin-overexpression mice (13 mice each group). The efficiency of human kallistatin gene transfer was detected by real-time qPCR analysis. Both in the spleen (**Figure S1A**) and dLNs (**Figure S1B**) of KS mice, mRNA levels of kallistatin were significantly increased compared to WT controls. The schematic diagram (**Figure 2A**) outlines the general procedure of disease induction and examination. We first tracked and recorded the development of EAU by indirect ophthalmoscope from day 10 to day 25 after immunization. As seen in **Figure 2B**, the clinical score of EAU in KS mice was significantly higher than WT mice. To observe EAU more objectively we then used retinal imaging and SD-OCT 14-17 days after disease induction. The representative fundus images obtained by retinal imaging showed that more severe vasculitis, linear or diffuse lesions were observed in the fundus of KS mice than WT mice (**Figure 2C**). The SD-OCT B-scan imaging correlated morphological features with inflammation; that is, more hyper-reflective foci of inflammatory cells in the vitreous cavity of KS mice. Accordingly, more retinal foldings and disruptions of the inner segment/outer segment (IS/OS) junction were seen in the KS mice (**Figure 2D**). Next, we quantitated the foci in the vitreous cavity using Image J and found that KS mice had significantly higher numbers of the foci than WT control mice (p = 0.0043, **Figure 2E**). These imaging results indicated that KS mice had increased choroidal focal or lineal chorioretinal lesions.

Pathological examination on day 21 after immunization was consistent with the *in vivo* ocular imaging results, showing a significant increased leukocyte infiltration and retinal folds in KS mice (**Figure 2F**). KS mice exhibited significantly higher scores compared to control mice (2.467 *vs* 1.429, **Figure 2G**). Collectively, all ocular imaging data and histopathological analysis demonstrated that kallistatin overexpression results in increased EAU severity.

### Overexpression of Kallistatin Results in Increased Th17 Infiltrates in the Eye and Lymphoid Organs During EAU

**Figure 3:**
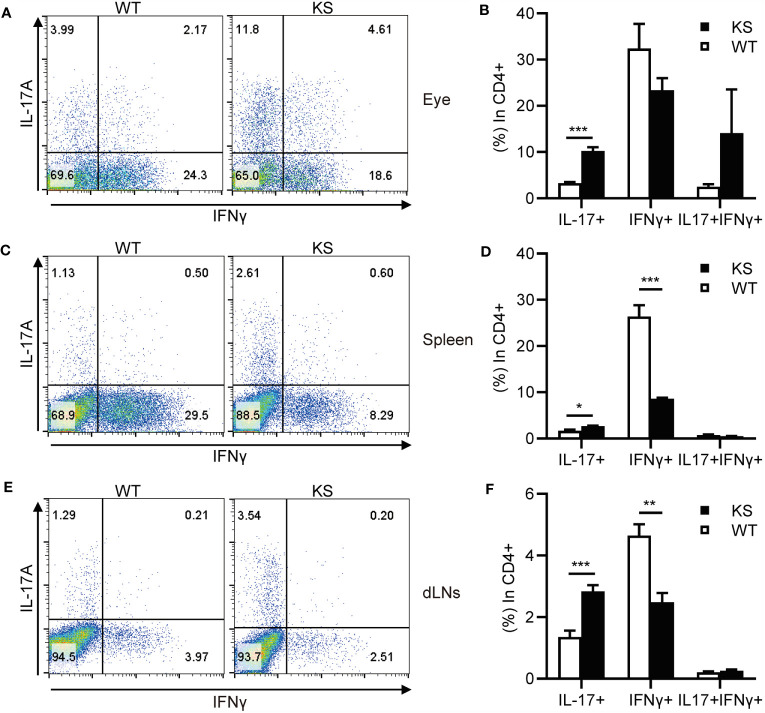
KS mice show an increased accumulation of autoreactive effector T cells and a skewing toward Th17 infiltration after EAU immunization. The cells harvested from eye, spleen or dLNs of WT and KS mice 14-16 days after immunization were stimulated with PMA, ionomycin plus brefeldin for 5 h, then stained with anti-CD4 mAb and intracellular anti-IL-17A and anti-IFN-γ mAbs, followed by FACS analysis. **(A, C, E)** FACS plot are representative of three similar independent experiments. **(B, D, F)** Bar diagram are shown for frequencies of IL-17A^+^, IFN-γ^+^ or IL-17A^+^IFN-γ^+^ cells in total CD4^+^ T cells from immunized WT and KS mice. Data are presented as mean ± SEM. *p < 0.05, **p < 0.01, ***P < 0.001. (Unpaired Student’s t test).

It’s been known that effector CD4^+^ T cells, especially Th1 and Th17 cells, play a pathogenic role in EAU development (1, 7, 31, 32). To determine whether kallistatin overexpression affects effector CD4^+^ T cells after EAU induction. We collected the cells from eyes, spleens and dLNs of WT and KS mice on day 14-16 post-immunization (p.i.) and stimulated with PMA/ion plus brefeldin for 5 h and analyzed the cells using flow cytometry. As shown in **Figures 3A–F**, the percentages of CD4^+^ IL-17^+^ (Th17) cells in the eyes and peripheral immune organs (spleen and dLNs) of KS mice were significantly higher than WT controls. The frequency of CD4^+^ IFN-γ^+^ (Th1) cells in the local eyes was comparable between KS mice and WT control mice (**Figures 3A, B**) and, the Th1 subset in the spleen and dLNs was significantly decreased in KS mice compared to WT mice (**Figures 3C–F**). Although CD4^+^ IL-17^+^ IFN-γ^+^ (Th17/Th1) subsets were observed in local eyes, there was no significant difference between WT and KS mice, and this subpopulation was detected at low levels (<1%) in both spleen and dLNs, as demonstrated in **Figure 3**. These results indicated that the overexpression of kallistatin affected pathogenic CD4^+^ T cells toward Th17 profile during EAU.

### Kallistatin Overexpression Promotes IRBP-Specific T Cell Proliferation

**Figure 4:**
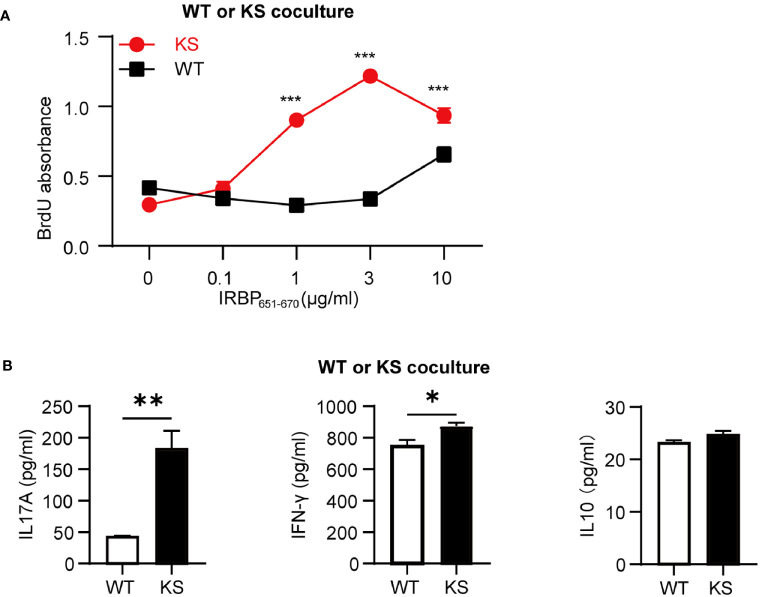
Kallistatin overexpression favors the proliferation of antigen-specific T cells. Responder T cells (TCs) and irradiated antigen presenting cells (APCs) isolated from spleen of WT or KS mice 14-16 days after immunization were stimulated with increasing concentrations of hIRBP_651-670_ antigen. Both the responder T cells and the irradiated APCs were from the same mice, either WT mice or KS mice. **(A)** The cell proliferation was measured after coculture for 60 h by incorporation of BrdU during the last 16-18 h of culture. **(B)** The supernatants of 10 μg/ml hIRBP_651-670_ peptide-stimulated coculture were collected, and the cytokine levels of IL-17A, IFN-γ and IL-10 were then determined by ELISA. Graphs are representative of two similar experiments (n = 3 mice per group). Data are shown as mean ± SEM. *p < 0.05, **p < 0.01, ***P < 0.001; **(A)** two-way ANOVA followed by a Sidak *post hoc* test; **(B)** Student’s t test.

To further determine that kallistatin overexpression affects IRBP-specific T cells, we measured the antigen specific responses of T cells from immunized KS and WT control mice. T cells and APCs from spleen of either WT or KS EAU mice were collected on day 14-16 p.i. and stimulated with increasing doses of the immunizing antigen. As shown in **Figure 4A**, T cells from immunized KS mice had an enhanced proliferative response, especially at 3 μg/ml of hIRBP_651-670_. Since both uveitogenic Th1 and Th17 cells are pathogenic for EAU induction (33, 34), we determined whether KS overexpression preferentially affected IFN-γ^+^ and/or IL-17^+^ autoreactive T cells. Supernatants from T cells stimulated with the highest concentration of IRBP_651-670_ (as in **Figure 4A**) were collected and cytokines of IFN-γ, IL-17A and IL-10 were determined by ELISA. As shown in **Figure 4B**, the amounts of IL-17 released into the culture supernatants by T cells from KS EAU mice were markedly higher than those produced by T cells from WT control mice. Although the levels of IFN-γ were significantly higher in KS cocultured system than those in WT system, the differences were small. Whereas IL-10 cytokines were similar between KS and WT EAU mice.

### Kallistatin Overexpression Directly Affects IRBP-Specific T Cells and Favors The Release Of IL-17A Production

**Figure 5:**
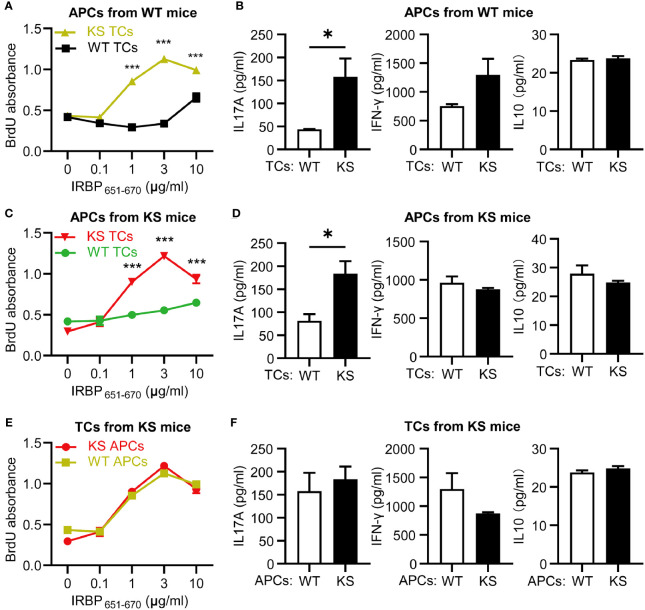
Kallistatin overexpression directly affects IRBP-specific T cells and favors the release of IL-17A production. T cells, either WT or KS, after 60 h in coculture with splenic APCs isolated from WT or KS mice. **(A, C, E)** Proliferation of responder T cells was determined by BrdU incorporation. **(B, D, F)** The supernatants in the presence of 10 μg/ml hIRBP_651-670_ peptide were collected, and levels of IL-17A, IFN-γ and IL-10 coproduction were measured by ELISA. **(A, B)** The responder T cells were from either WT or KS mice, while the irradiated APCs were from WT mice. **(C, D)** T cells from WT or KS mice were cocultured with APCs from KS mice. **(E, F)** Irradiated APCs from WT or KS mice were cocultured with T cells from KS mice. Graphs are representative of two similar experiments (n=3 animals per group). Data are shown as mean ± SEM. *p < 0.05, ***P < 0.001; **(A, C, E)** two-way ANOVA followed by a Sidak *post hoc* test; **(B, D, F)** Unpaired Student’s t test.

To distinguish whether the high responsiveness of autoreactive T cells in KS animals resulted from dysfunction of autoreactive T cells or APCs, we performed cross-tests in which T cell proliferation was measured using several combinations of responder T cells and APCs from IRBP_651-670_-immunized KS or WT control mice. T cells from KS mice showed higher responses to increasing doses of immunizing antigen in the presence of APCs from either KS or WT control mice, whereas T cells from control mice reacted well but lower than KS mice in the presence of APCs from control mice and KS mice (**Figures 5A, C**), indicating that overexpressing KS directly affects T cell hyperresponsiveness in KS mice. In addition, the levels of IL-17A secreted by KS-derived T cells were higher than those WT-derived T cells, whereas the levels of IFN-γ and IL-10 released were not significantly different (**Figures 5B, D**). However, when the autoreactive T cells were from KS mice, and APCs were derived either from WT or KS, there was no significant difference in T cell proliferation and three cytokine productions between the two coculture systems (**Figures 5E, F**). These results suggest that kallistatin overexpression is associated with the enhanced reactivation of IRBP-specific T cells, especially favoring Th17 cells.

### Overexpressing Kallistatin Favors Th17 Cell Differentiation

**Figure 6:**
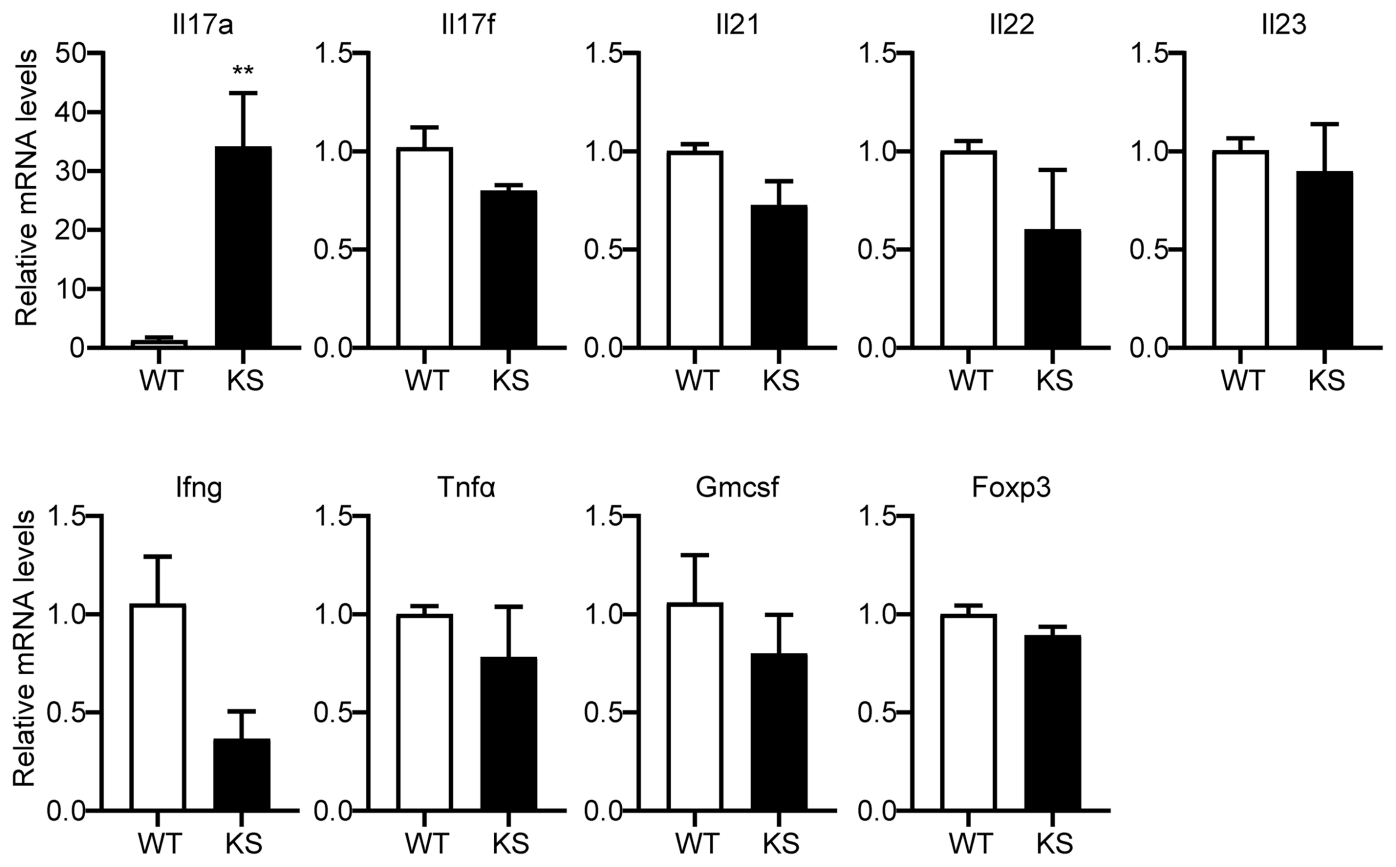
Kallistatin overexpression favors mRNA expression of Il17a in CD4^+^ T cells. The total CD4^+^ T cells were sorted from spleens of unimmunized (naïve) WT or KS mice using a mouse CD4^+^ T cell enrichment kit. Quantitative real-time PCR analysis was performed to determine the relative mRNA expression levels of *Il17a, Il17f, Il21, Il22, Il23, Ifng, Tnfα, Gmcsf* and *Foxp3*. The gene levels were normalized using *Gapdh*, and the level of each gene was expressed as a ratio of WT group. Data are mean ± SEM (n = 3-5 mice). **p < 0.01. (Unpaired Student’s t test).

To further investigate the effect of kallistatin on Th17 cells, we first isolated CD4^+^ T cells from spleen of WT and KS mice, and identified the genes differently expressed in T cell subsets. As shown in **Figure 6**, only Il17a mRNA in CD4^+^ T cells derived from KS mice was significantly higher than WT mice, whereas the expressions of other mRNAs, including *Il17f, Il21, Il22, Il23, Ifng, Tnfα, Gmcsf* and *Foxp3* were not significant different between KS and control mice, indicating that overexpressing kallistatin might only regulate IL-17A expression.

**Figure 7:**
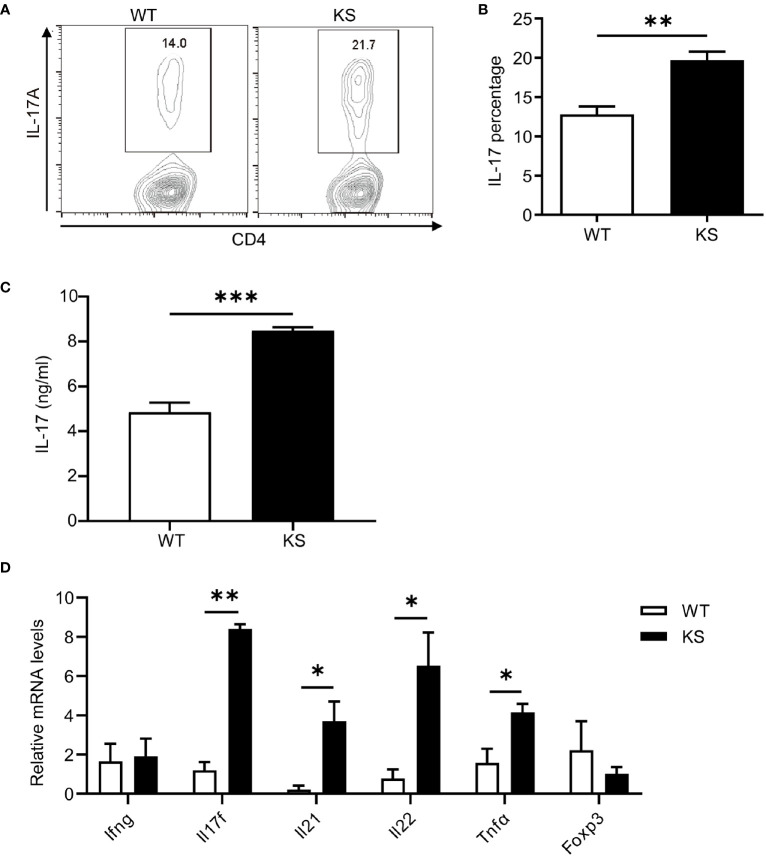
Kallistatin favors differentiation of CD4^+^ naïve T cells to effector Th17 cells. Naïve CD4^+^ T cells purified from spleen and dLNs in WT and KS mice and cultured under Th17 polarizing conditions. **(A)** On day 5 of differentiation, the cells were then stimulated with PMA, ionomycin and brefeldin for 5 h, followed by flow cytometry analysis. **(B)** Bar diagram is shown for IL-17A^+^ frequencies in CD4^+^ T cells of WT and KS mice. **(C)** The supernatants harvested at 48 h of culture were assessed for IL-17A by ELISA. **(D)** Cells collected after 48h culture were determined for the mRNA expression of indicated cytokines by real-time qPCR analysis. Data are representative of three individual experiments and graphs are shown as mean values ± SEM. n = 3, *p < 0.05, **p < 0.01, ***P < 0.001. (Unpaired Student’s t test).

We then assessed the potential contribution of kallistatin to Th17 differentiation. Naïve CD4^+^CD25^-^62L^hi^CD44^lo^ T cells sorted from the spleen and dLNs of KS or WT mice were cultured under Th17 polarization condition for 5 days. As shown in **Figures 7A, B**, compared with WT mice, differentiated T cells from KS mice had significantly elevated percentages of IL-17A-expressing cells. We also determined the levels of IL-17A cytokine in the supernatants, which were significantly higher than controls (**Figure 7C**). In addition, we examined the expression of inflammatory cytokines under Th17 polarized conditions. A remarkable increase in mRNA levels of *Il17a*, *Il17f* and *Il22*, *Il23*, but not *Ifng* and *Foxp3*, was observed in naïve T cells from KS mice (**Figure 7D**). These data together indicate that overexpressing kallistatin boosts Th17 polarization.

### Kallistatin Overexpression Has No Effects on Activation and Proliferation of T Cells From Naïve Mice

**Figure 8:**
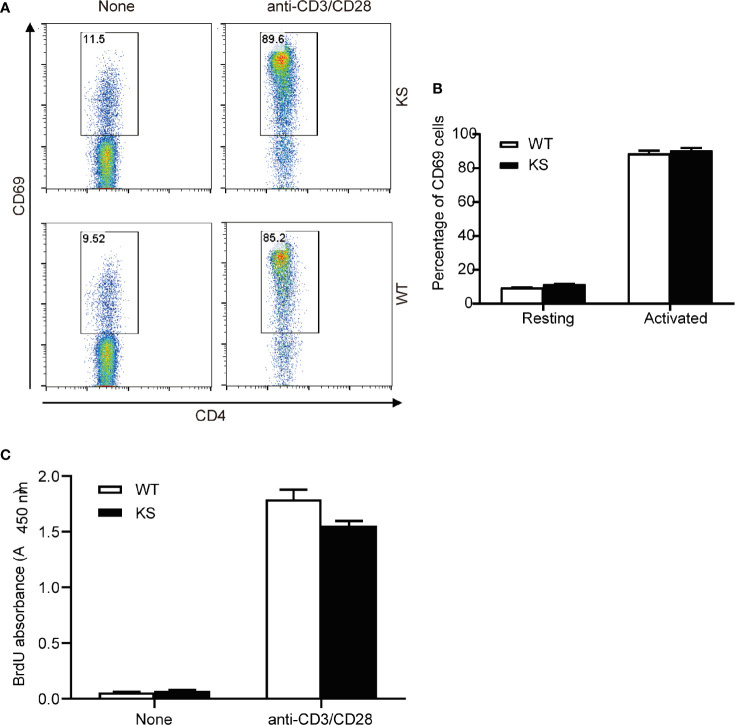
Effects of Kallistatin overexpression on T cell activation and proliferation of naïve mice. CD4^+^ T cells purified from naïve WT and KS mice were stimulated without (None) or with anti-CD3 and anti-CD28 mAbs. **(A, B)** After stimulation for 5 h, cells were collected and washed, followed by staining for CD69 and flow cytometry analysis. Representative FACS plot **(A)** and bar diagram **(B)** for frequencies of CD69^+^ T cells are shown. **(C)** After stimulation for 24 h, the proliferation of T cells was detected by BrdU incorporation. Data are representative from three independent experiments and shown as mean ± SEM. (Unpaired Student’s t test).

To assess the effects of kallistatin on CD4^+^ T cells from naïve mice, we purified CD4^+^ T cells from spleens and dLNs of WT or KS mice, and incubated them in the presence of anti-CD3 and anti-CD28 antibodies (mAbs). After 5 h, the activation status of CD4^+^ T cells was evaluated by flow cytometry with the mAb against CD69, a marker of early T cell activation. We found that WT and KS mice had similar CD69 levels in the CD4^+^ T cells, both in the resting and activated status (**Figures 8A, B**). In addition, proliferative responses of CD4^+^ T cells to CD3/CD28 stimulation were not different between WT and KS groups determined by BrdU incorporation assay (**Figure 8C**). These data suggest that overexpressing kallistatin doesn’t affect the activation and proliferation of CD4^+^ T cells from naïve mice *in vitro*.

## Discussion

It has been reported that kallistatin exhibits various functions in angiogenesis, oxidative stress and inflammatory activities (21, 23, 24). However, much less attention has been paid to the role of kallistatin in the pathogenesis of autoimmune diseases. In this study, we identify the highly expressed kallistatin protein in VKH patients for the first time. Furthermore, we establish an association between overexpressing kallistatin and a mouse model of autoimmune uveitis, that is, KS mice are more susceptible to EAU induction. Overexpressing kallistatin promotes Th17 polarization *in vivo* and *in vitro*. Accordingly, there is a dominant Th17 subset in diseased eyes, and upregulated antigen-specific responses of Th17 cells in immunized KS mice. These observations support a concept that a high level of kallistatin promotes adaptive responses to uveitis by enhancing differentiation of CD4^+^ T cells towards Th17 effector cell lineage. A critical finding of our study is that overexpressing kallistatin is directly responsible for the proliferation and IL-17A production of hIRBP_651-670_–specific CD4^+^ T cells, leading to an aggravated uveitogenic phenotype.

Our data show that kallistatin levels are elevated in VKH patients compared to those non-uveitis subjects, which raises the intriguing possibility that kallistatin might be involved in the regulation of inflammation of uveitis. We further reveal that transgenic elevation of human kallistatin in mice contributes to developing EAU. In contrast to our findings of EAU in KS mice, EAU in KS mice immunized with IRBP_1-20_ peptide was reported to be milder than WT mice (35). The contradictory results might be attributable to the different animal models and cell types. One of major differences is that the IRBP peptides used for generation of EAU are different between two labs. We used hIRBP_651-670_, whereas the other lab used IRBP_1-20_. It has been reported that different IRBP peptides and model-induced methods elicit different uveitogenic and immunological responses (1, 8). IRBP_651-670_ elicits severer EAU disease and specific T cell responses than IRBP_1-20_ in B6 mice with the H-2b haplotype (36). We further evidenced that severe EAU was induced by IRBP_651-670_ in KS mice, supported by their high responses of IRBP-specific T cell proliferation and elevated Th17 cell generation. Additionally, Fauziyya et al. was more interested in Treg cells in the resolution and post-EAU phases (35), while we mainly focused on Th1 and Th17 inflammatory cells at onset and peak phases of the disease. In their study (35), neither the effects of overexpressing kallistatin on Th1 or Th17 cells in EAU nor the mechanisms of kallitatin inhibiting EAU have been studied. These results may reflect diverse characteristics of kallistatin in different models and possible different activities depending on the stage of the disease. In any cases, additional studies are needed to validate these hypotheses.

Accumulating evidence suggests that CD4^+^ T cells differentiate into functionally distinct subpopulations, protective or pathogenic, and mediate the balance of the immune responses (14, 37). It’s been strongly supported that either a Th17 or a Th1 effector response can drive EAU depending on the animal model (8, 34, 38, 39). We demonstrated here that compared to WT mice, Th17 infiltrates at peak stage of a monophasic EAU model were increased in the eye and peripheral lymphoid organs of KS mice. In contrast, Th1 subset was significantly decreased in the spleen and dLNs of KS mice but not in the eye. This difference could be as a result of the different milieu of secondary lymphoid organs and local eye. Additionally, we did not observe that overexpressing kallistatin impacted the IFN-γ^+^ IL-17A^+^ subset or Foxp3^+^ Treg subset (data not shown) during EAU. These data may explain why KS mice developed severe EAU, as the overexpression of kallistatin promotes autoimmune inflammation by providing a better setting for effector Th17 instead of Th1 cells in the local tissues. Previous study has also demonstrated that Th1 cytokine such as IFN-γ inhibits the development of Th17 (15).

The proliferative and pathogenic analyses of EAU splenocytes suggest that kallistatin overexpression contributes to the reactivation of autoreactive T cells in response to IRBP_651-670_ antigen. Meanwhile, we have demonstrated that IRBP_651-670_-specific T cells, but not APCs, from KS mice exhibit a more activated and aggressive phenotype. In line with this, kallistatin favors IRBP_651-670_-reactive T cells releasing IL17A, but not IFN-γ or IL10. These suggest that autoreactive T cells, especially Th17 cells play a central role in controlling accelerated inflammation of KS mice during EAU. This affirmation is supported by the work of Dody et al, who states that IFN-γ is not necessary for the pathogenesis of EAU (40).

Our mRNA data of purified T cells show a higher level of IL17A in unimmunized KS mice than that in naïve WT mice, confirming the critical role of kallistatin in a pro-Th17 cytokine milieu, which may be mainly related to the expression of IL17A. Although IL-17A and IL-17F are highly homologous, they perform distinct functions: IL-17A plays important roles in inflammation, autoimmunity, and host defenses against bacterial and fungal infections; whereas IL-17F is mainly involved in mucosal host defense mechanisms (41). Furthermore, under Th17-polarizing conditions, KS naïve CD4^+^ T cells showed a higher percentage of IL17A^+^ populations, and increased mRNA levels of Th17 signature cytokines, such as IL17A, IL17F, IL21 and IL22. Despite the addition of various stimuli factors under *in vitro* polarized conditions, the *in vivo* and *in vitro* milieus are different, the *in vivo* regulatory mechanisms are relatively complex and more unclear. This may explain why only IL-17 was elevated and neither IL-21 nor IL-22 was elevated in our vivo mRNA data. These data together suggest that kallistatin gives rise to a high differentiation of naïve T cells into Th17 cells. In addition, our data showed that kallistatin did not activate the T cells of naïve mice by CD3/CD28 mAbs *in vitro*, suggesting that increased kallistatin helps to promote antigen-specific T cells during the development of autoimmunity. However, the detailed mechanism by which kallistatin regulates Th17 cells remains to be further clarified.

In conclusion, the results of our study demonstrate that overexpressing kallistatin favors Th17 generation which promotes autoimmune responses. We first demonstrated that kallistatin was highly expressed in VKH patients. In EAU, high levels of kallistatin promoted Th17 polarization, and increased proportions of CD4^+^IL17A^+^ cells in local eye and peripheral immune organs, thereby aggravating the intraocular inflammation. These findings provide an insight into the roles of kallistatin in Th17 polarization and excessive inflammatory reactions in EAU. Hence, modulating kallistatin levels and reducing Th17 cells might be a potential therapeutic strategy for Th17 mediated autoimmune diseases.

## Data Availability Statement

The raw data that supports the conclusions of this article will be made available by request to the corresponding author, without undue reservation.

## Ethics Statement

The studies involving human participants were reviewed and approved by Ethics Committee of Tianjin Medical University Eye Hospital (Tianjin, China). The patients/participants provided their written informed consent to participate in this study. The animal study was reviewed and approved by Animal Care and Use Committee, Tianjin Medical University.

## Author Contributions

XZ, HS, and NC designed the research and interpreted data. J-XM Lab generated and provided the transgenic mice. NC, SC, XC, ZZ, and LW performed experiments and analyzed data. KLG mated and bred the mice. NC wrote the manuscript. HS and XZ reviewed the manuscript. All authors contributed to the article and approved the submitted version.

## Funding

This work was supported by National Natural Science Foundation of China (81671642, 81870651), Tianjin Science and Technology Support Plan (20YFZCSY00990), and Natural Science Foundation of Tianjin (20JCZDJC00100).

## Conflict of Interest

The authors declare that the research was conducted in the absence of any commercial or financial relationships that could be construed as a potential conflict of interest.

## Publisher’s Note

All claims expressed in this article are solely those of the authors and do not necessarily represent those of their affiliated organizations, or those of the publisher, the editors and the reviewers. Any product that may be evaluated in this article, or claim that may be made by its manufacturer, is not guaranteed or endorsed by the publisher.
