# Supplementary material for: Overexpressing Kallistatin Aggravates Experimental Autoimmune Uveitis Through Promoting Th17 Differentiation
Source: Front Immunol. 2021 Oct 18;12:756423. doi: 10.3389/fimmu.2021.756423 (PMC8558411; doi:10.3389/fimmu.2021.756423)
Supplement: Supplementary file 1 [file DataSheet_1.docx]

**Supplementary Figures**


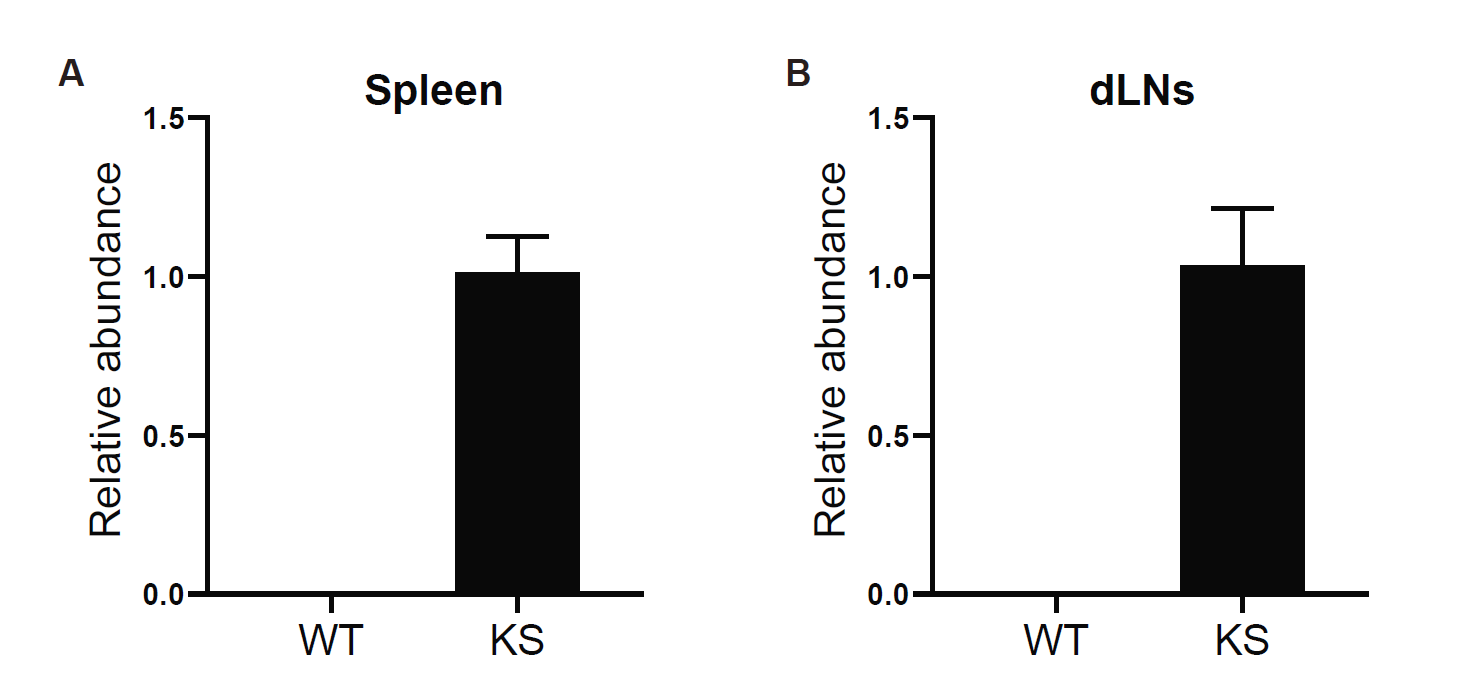
**Figure S1. The expression of human kallistatin gene in the transgenic mice.** The spleen and dLNs were isolated from naïve WT or KS mice. Expression of human kallistatin mRNA (transgenic) were extracted from the spleen (A) and dLNs (B) of mice and assessed by RT-qPCR using genotyping primers (*SERPINA4*). The gene levels were normalized using *Gapdh*, and the level of Kallistatin gene was expressed as a ratio of KS group. Data are mean ± SEM (n=3 mice per group).
